# Community-Based Participatory Obesity Prevention Interventions in Rural Communities: A Scoping Review

**DOI:** 10.3390/nu16142201

**Published:** 2024-07-10

**Authors:** Saagar Dhanjani, Haley Allen, Beatriz Varman, Chishinga Callender, Jayna M. Dave, Debbe Thompson

**Affiliations:** 1 Department of Natural Science, Rice University, Houston, TX 77005, USA; saagar.dhanjani@gmail.com (S.D.); hcallen122@gmail.com (H.A.); 2 The Texas Medical Center Library, Houston, TX 77030, USA; beatriz.varman@library.tmc.edu; 3 USDA/ARS Children’s Nutrition Research Center, Department of Pediatrics, Baylor College of Medicine, Houston, TX 77030, USA; chishinga.callender@bcm.edu (C.C.); jmdave@bcm.edu (J.M.D.)

**Keywords:** community-based participatory research, school-based obesity prevention, community-engaged research, rural obesity

## Abstract

Child obesity is a worldwide public health concern. In America, children from rural areas have greater odds of obesity in comparison to those from urban areas. Community-engaged research is important for all communities, particularly under-represented communities. This paper reports the results of a scoping review investigating community-engaged research in obesity prevention programs tested with school-aged children in rural America. A literature search of Medline Ovid was conducted to identify interventions reporting the results of obesity prevention interventions that promoted a healthy diet or physical activity (PA) behaviors to school-age children in rural communities of the United States (US). After title and abstract review, potentially relevant citations were further examined by assessing the full text. Each stage of review was conducted by two independent reviewers. Twelve studies met the inclusionary criteria and are included in this review. Most of the studies focused on elementary school participants (n = 7) and improving both diet and PA (n = 9). Out of the twelve studies, only five included the target audience in intervention development or implementation. The most popular type of community engagement was community participation (n = 4). This review revealed that community-engaged research is under-utilized in obesity prevention interventions tested with school-aged children in rural US communities.

## 1. Introduction

Obesity among youth is an area of increasing concern. Globally, the prevalence of childhood overweight and obesity increased from 8% in 1990 to 20% in 2020 [1]. Similarly, in the United States (US), the country in which the research reported in this paper was conducted, childhood obesity prevalence among 2–19-year-olds was 19.7% in 2017–2020 [2]. Further, US data reveal that obesity prevalence is not equally distributed among all groups. Although racial and ethnic disparities are well documented [2,3], geographic disparities also exist between rural and urban areas, especially among teens. This is an emerging crisis. As of 2016, 19% of the US population and 13 million children lived in rural areas [4]. In 2020, 20% of the US population was classified as rural [5], making rural obesity an important public health issue. This concern extends far beyond US borders, with 44.7% of the world’s population residing in rural areas in 2018 [6].

A meta-analysis from 2015 found that children living in rural US communities have 26% greater odds of obesity in comparison to children living in urban areas [7]. More recent research confirmed this disparity, although regional variability was observed [8], indicating a need for targeted intervention approaches to reflect the needs of the community. Although the risk of obesity is influenced by numerous factors, two that are under volitional control are diet and physical activity (PA) [9]. While many studies have reported higher levels of PA among rural children with obesity compared to their urban counterparts [10,11,12,13,14,15,16], others have reported minor differences in dietary intake [17,18]. For instance, Euler et al. showed a higher intake of whole grains, potatoes, and added sugar with every 1-unit increase in log population density [18], and Liu et al. found that rural adolescents (12.2%) had a slightly lower intake of daily fruit compared to their urban counterparts (16.5%) [12]. The discrepancy between the higher prevalence of obesity among rural adolescents despite higher PA levels and similar dietary intake demonstrates the need for obesity prevention programs tailored to youth living in rural communities.

A recent scoping review examining school-based obesity prevention programs in rural communities found that most studies focused on children in elementary or middle schools, often integrating both PA and nutrition [19]. However, the review did not report stakeholder or community involvement in intervention conceptualization, implementation, and/or evaluation. This is a significant oversight, given that working with the community to develop and/or adapt an intervention is an important component of intervention research [20]. This approach involves the community in one or more phases of the research and can lead to important insights regarding the topic of interest [21]. A meta-analysis concluded that community engagement in research was effective at improving health outcomes, particularly in under-represented groups [22]. Therefore, community engagement has been identified as an essential component of interventions, particularly those designed to achieve health equity in obesity prevalence.

While various terms describe community participation in research, “community-engaged research” is the term used throughout this paper [23]. Community-engaged research involves collaboration between community members and researchers to develop, implement, and/or evaluate an intervention [24,25]. Key et al. [26] demonstrated that community engagement occurs at different levels, ranging from no involvement to community-driven initiatives, with increased involvement linked to increased long-term adherence, satisfaction, and acceptance [27]. Given rural populations’ historically low participation rates in research [28], community-engaged research may be especially important for enhancing intervention engagement and effectiveness in rural communities. This review thus explored community engagement in obesity prevention interventions targeting school-aged youth living in rural communities in the US. 

## 2. Materials and Methods

This scoping review specifically examines community engagement and target audience involvement in obesity prevention interventions developed and/or tested with school-aged children in rural communities in the US. Guided by the framework developed by Key et al. [26], the research questions for this review were as follows: (1) To what extent were stakeholders engaged in the research process?; and (2) How were the target audience (Kindergarten—12th graders) and/or their parents/caregivers involved in the research? 

### 2.1. Data Sources

This review followed the Preferred Reporting Items for Systematic Reviews and Meta-Analyses (PRISMA) adapted for scoping reviews [29].

A comprehensive literature search was conducted on Medline Ovid by a professional research librarian located at the Texas Medical Center library. The search was designed to identify interventions promoting obesity prevention, healthy diet, or physical activity behaviors to school-age children living in rural US communities. MeSH headings were utilized (Obesity, Morbid or Overweight, Weight Loss, Child, Adolescent, Pediatric Obesity, Rural Health, Hospitals, Rural, Rural Population, or Rural Health Services, Primary Prevention, Health Promotion, Early Medical Intervention, Psychosocial Intervention, Internet-Based Intervention, exp Clinical Study), along with corresponding keywords, phrases, and truncated terms. Subsequently, the search strategy was then translated to Embase (Elsevier), PsycInfo (Ovid), Cochrane Library (Wiley) and Cumulated Index in Nursing and Allied Health Literature (CINAHL) Plus with Full Text (EBSCO). The detailed search strategy is outlined in Table S1.

### 2.2. Data Collection Procedures

The searches were conducted on 9–14 December 2021. A total of 2712 citations were identified on the subject matter. These citations were combined into an EndNote library and de-duplicated among themselves, resulting in a total of 1513 unique citations.

The inclusionary criteria for the initial review were as follows: the study must be interventional, participants must have been healthy at the start of the intervention, at least 75% of participants were between the ages of 5 and 18 years old, and at least 75% of participants lived in a rural community. Additionally, the study had to be conducted in the US and focused on obesity prevention. Conference abstracts, theses, dissertations, and proceedings were excluded. Although there is no consensus definition, rural communities are generally defined as any area that is not considered urban [30]. This review included any article conducted in an area defined as rural by the authors.

**Figure 1:**
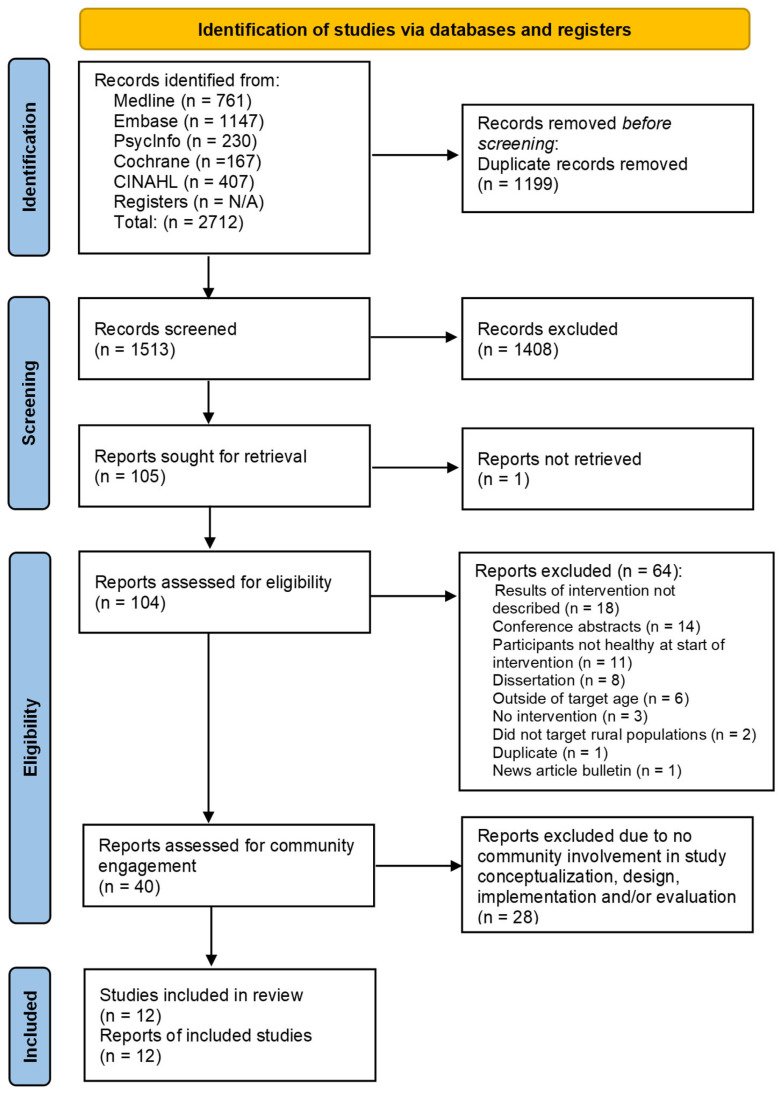
PRISMA 2020 flow diagram for search results and screening outcomes.

This review was conducted in four stages, with articles excluded after each stage, as shown in Figure 1. Two reviewers independently conducted each stage of the review, adhering to the inclusionary criteria. Regular meetings were held to compare the results and discuss any discrepancies.

In the first stage, articles were categorized into three groups based on abstract and title review: “Yes”, “No”, and “Maybe”. In stage 2, “No” articles were excluded, “Yes” articles were retained, and “Maybe” articles were assessed using the full article to determine inclusion. At the conclusion of stage 2, 40 articles met the inclusionary criteria. Because of the expanded focus on community-engaged research, articles meeting the inclusionary criteria were further reviewed (stage 3) to identify those that reported sufficient details on community engagement to enable reviewers to determine what research was conducted, with whom, and how. Articles lacking community involvement in study conceptualization, design, implementation, and/or evaluation, or those providing insufficient detail to assess community involvement, were excluded. At the conclusion of this stage, 12 articles were determined to provide sufficient information to address the research questions.

In stage 4, two reviewers independently extracted information from the 12 articles regarding study, intervention, and community engagement characteristics. A data extraction sheet that included definitions and extraction categories was created. Reviewers independently extracted the information and routinely met to compare extractions and resolve discrepancies. Extraction characteristics included the intervention’s name, geographic location, definition of rurality, sample size, focus, goals, target audience, research design, duration, delivery location, components, theoretical frameworks employed, and research outcomes. Additionally, the implementer, level of community involvement in study design using the Key et al. framework [26] and socio-economic status (SES) of the target population were noted.

Interventions with study characteristics and outcomes published separately were cited individually but considered a single study. Consequently, one study may have multiple citations.

## 3. Results

The twelve articles that met the inclusionary criteria are characterized and described below.

### 3.1. Study Characteristics

This section provides an overview of the study characteristics, including geographic region, SES, research design, and target audience (Table 1). Studies were conducted in rural counties spanning the US, ranging from California to North Carolina. Only two states, Colorado [31,32,33] and Kentucky [34,35,36], reported more than one study. Five studies [34,35,36,37,38,39,40] were conducted in states comprising the Appalachian Region, an economically disadvantaged area of the country [41].

SES was defined as a measure of income in the target population. Out of the twelve studies included, nine reported on the SES of their target population. Among these, five utilized the percentage of students receiving free/reduced-price lunch as an indicator of SES [31,32,33,34,38,42]. The remaining three studies relied on income to describe SES [37,39,40,43,44].

The most popular study design was a quasi-experimental, one group design (n = 5) [34,38,39,40,42,45], followed by three studies employing a quasi-experimental design with both intervention and control groups [43,44,46,47,48]. Additionally, two studies used a randomized two-group design [35,36,37]; one utilized a pair randomized design [32,33]; and one adopted a three-group quasi-experimental study design [31].

Seven studies included elementary school participants, defined as students in grades 1–5 [31,32,33,34,37,39,40,43,44,46]. Two studies were conducted with middle school students, defined as students in grades 6–8, or between the ages of 11 and 14 [42,45]. Furthermore, two studies targeted high school students, defined as students between grades 9 and 12, or between the ages of 14 and 18 [35,36,38]. One study included children between the ages of 8 and 12, which fell between our two categories of elementary school and middle school students [47,48].

Rurality was not clearly defined in most studies. Only one provided clarification regarding its definition of rurality. Hawley et al. [45] used federal population density guidelines established in 2003 to recruit rural participants. Additionally, one out of the six schools recruited in the Askelson et al. study [42] was located in an urban county.

**Table 1:** Study characteristics.

Author(s) (Year of Publication)	Intervention Name	Geographic Location	Socio-Economic Status	Research Design	Target Audience
Askelson, N.M., et al. (2019) [42]	None	Iowa	28.5% of students received free/reduced-price lunch	Quasi-experimental (pre/post—one group)	Middle School Students
Belansky, E.S., et al. (2006) [31]	INPAP	Colorado	67% of students were eligible for free/reduced-price lunch	Quasi-experimental—3 groups	2nd–3rd-grade students
Belansky, E.S., et al. (2013) [33]; Belansky, E.S., et al. (2009) [32]	School Environment Project	Colorado	An average of 69% of students at each school received free/reduced-price lunch	Pair randomized design: 5 schools receiving the AIM intervention and 5 schools receiving the SHI intervention	Elementary school students
Canavera, M., et al. (2008) [34]	None	Kentucky	An average of 32% of students at each school received free/reduced-price lunch	Quasi-experimental (pre/post—one group)	5th-grade students
de la Torre, A., et al. (2013) [43]; Sadeghi, B., et al. (2019) [44]	Niños Sanos, Familia Sana	California	58.9% of students were below the poverty line	Two groups (intervention and control)—quasi-experimental	3–8-year-old children of Mexican origin
Donnelly, J.E., et al. (1996) [46]	None	Nebraska	Not reported	Two groups (intervention and control)—quasi-experimental	3rd–5th-grade students
Greening, L., et al. (2011) [37]	TEAM Mississippi	Mississippi	Treatment group median income: USD 30,713 Control group median income: USD 29,904	Two groups (intervention and control)—randomized	6–10-year-old children
Gustafson, A., et al. (2017) [35]; Gustafson, A., et al. (2019) [36] for outcomes	Go Big and Bring it Home	Kentucky and North Carolina	Not Reported	Two groups (intervention and control)—randomized	14–16-year-old adolescents
Hawley, S.R., et al. (2006) [45]	Pilot Community Prevention Program	Kansas	Not reported	Quasi-experimental (pre/post—one group)	6th-grade students
Lynch, W.C., et al. (2012) [44]; Eldridge, G., et al. (2016) [43]	4-Health	Montana	Not Reported	Two groups (intervention and control)—quasi-experimental	Families with 8–12-year-old children
Schetzina, K.E., et al. (2009) [39]; Schetzina, K.E., et al. (2009) [40]	Winning with Wellness	Tennessee	More than 50% of students were economically disadvantaged	Quasi-experimental (pre/post—one group)	3rd–4th-grade students
Smith, L.H., et al. (2014) [38]	Sodabriety	Ohio	40% of students received free/reduced-price lunch	Quasi-experimental (pre/post—one group)	9th–12th-grade students

### 3.2. Intervention Characteristics

This section describes the characteristics of the interventions, including the focus, delivery method, duration, components, theoretical framework employed, and implementers.

Out of the twelve studies included in the review, three focused on improving diet only [35,36,38,42] and nine focused on diet and PA [31,32,33,34,37,39,40,43,44,45,46,47,48]. No studies focused exclusively on PA.

All interventions were delivered at school with the exception of two, with one being a virtual intervention [31,32,33] and one being delivered at a County Extension Office [47,48]. The intervention duration ranged from 1 month [38] to 3 years [43,44]. Nine out of the twelve studies were at least the length of one academic semester [31,32,33,35,36,37,39,40,42,43,44,46,47,48].

The most common intervention across the 12 studies was educational sessions promoting PA through programs or school activities and nutritional enhancements through changes to school menus. Nine studies included educational sessions or dissemination of material on a variety of topics such as nutrition, PA, diabetes, goal setting, self-efficacy, and stress management [31,34,37,38,39,40,43,44,45,46,47,48]. Six studies promoted PA through events or activities [31,37,39,40,43,44,45,46], and three studies included changes in school menus [39,40,42,46]. Other intervention components included replacing deep-frying equipment with ovens, providing kits with water bottles, magnets, bookmarks, and T-shirts, encouraging healthy food purchases, providing families with a fruit/vegetable voucher, promoting health and wellness lectures for teachers and staff, offering free health screenings, and home visits by a family advisor [31,35,36,37,38,39,40,43,44].

In the design and development of the interventions, various theories and models were employed. Six studies used the Social Cognitive Theory [31,32,33,34,35,36,43,44,47,48], while one study applied the Social Learning Theory [37], and another incorporated the principles of behavior change [45]. Additional theories/models included the transtheoretical model [45], behavioral economics [42], the “health at every size” approach [47,48], the social marketing theory [47,48], the coordinated school health model [39,40], the health belief model [43,44], and the Piaget cognitive development theory [31]. Although most studies identified a theoretical framework, only four studies indicated how theory informed the intervention content or design [34,35,36,42,47,48].

A diverse range of personnel were involved in program implementation. Classroom teachers were utilized in six studies [18,34,38,39,40,43,44,46], students were utilized in three studies [35,36,38,42], and foodservice staff were employed in two studies [42,46]. School administration played a role in two studies [32,33,39,40], and parents or family advisors in two studies [31,39,40]. Other contributors included dietitians [37], educators from the Department of Education [37], physical education teachers [34], external facilitators [32,33], the University of California Cooperative Extension [43,44], and county agents [47,48]. More specific information on intervention focus (diet, PA, both), intervention setting, duration, components, theoretical framework, and implementer can be found in Table 2.

The 12 articles were subsequently categorized according to the Key et al. continuum [26]. The first category “no community involvement” was excluded due to the nature of the research question. The next six categories included “community informed”, “community consultation”, “community participation”, “community initiated”, “community based participatory research”, and “community driven/led”.

In the “community informed” category, the researchers gathered insights from the community to guide and inform the intervention. In the “community consultation” category, the community provided feedback and advice on the proposed research plan. In the “community participation” category, community members were actively involved with the research process such as assisting in recruitment efforts and serving on advisory committees. In the “community initiated” category, the researchers responded to the community needs but without direct community involvement in the design or analysis of the research. In the “community based participatory research (CBPR)” category, community participation was emphasized in every stage of the research process. Finally, in the “community driven” category, the community started and led the research, seeking researcher support.

**Figure 2:**
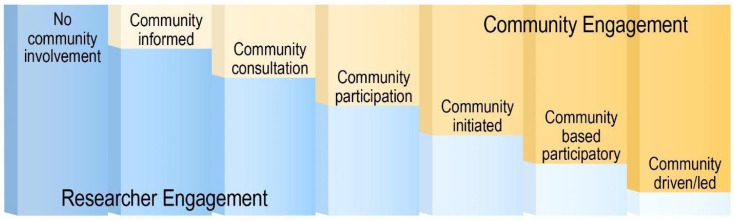
Different levels of community and research engagement in research. Adapted from Key et al. [26].

Examples for each of the remaining six levels are presented in Table 3 and a figure representing the balance between researcher and community involvement is presented in Figure 2. 

### 3.3. Reported Outcomes

Seven studies examined changes in anthropometric measures, including body mass index (BMI), body fat percentage, or weight [35,36,37,39,40,43,44,45,46,47]. However, only two reported significant improvements in one of these categories at the final follow-up [37,43,44]. Conversely, studies showed positive changes in dietary behaviors, with significant improvements observed in foods served in cafeterias [32,33,39,40,42,46], nutrition knowledge [31,37,46], water consumption [34,38], and attitudes towards nutrition [31]. While five studies investigated dietary intake, such as fruit/vegetable consumption and fat/sodium intake [34,35,36,37,45], three reported significant improvements [35,36,37,46]. Similarly, only one study each reported significant reductions in sugar-sweetened beverage consumption [38] and improvements in physical fitness [37] out of three [34,35,36,38] and two studies [37,46] investigating these outcomes, respectively. The reported significance of these outcomes reflects their status at the final follow-up time point. 

### 3.4. Review Question #1: To What Extent Were Stakeholders Engaged in the Research Process?

For the purposes of this review, “stakeholders” were defined as community members involved in intervention development, adaptation, and/or implementation. The “target audience” (i.e., those for whom the intervention was designed to affect behavior) was defined as school-aged children living in rural US communities. Therefore, to explore this question, we identified various stakeholders involved in the research, focusing on school-aged children (level 1), followed by their caregivers (e.g., parent or caregiver) (level 2), intervention implementers (e.g., teachers or school staff if school-based) (level 3), and/or the larger community (e.g., broader community members not included in levels 1–3 (level 4). Stakeholders were categorized by varying levels to capture the degree to which members of the target audience themselves or their parents were included in the research. These can be seen in Table 4. 

Each article included in this review was placed in one of the Key et al. levels of community and research engagement categories [26]. The most frequent level of community engagement was community participation, where four out of the twelve studies actively involved community members in the research process, such as providing feedback on the proposed intervention, developing/modifying the intervention material, and delivering the intervention [31,39,40,43,44,46]. Three studies were classified as CBPR, where stakeholders collaborated in the planning, implementation, and evaluation of the intervention [32,33,38,42]. Additionally, three studies fell into the community consultation category where feedback and advice were sought, including insights into barriers that need to be addressed and current behavioral patterns [34,45,47,48]. Two studies were classified as community-informed, utilizing surveys and focus groups to gather input on proposed research and baseline characteristics [35,36,37].

Table 5 displays these levels for each article along with more specific details regarding stakeholder involvement in the participatory design, utilization of the target audience or stakeholders in the development of the intervention, and the stakeholder level. 

### 3.5. Research Question #2: How Were the Target Audience (K-12th Graders) and/or Their Parents/Caregivers Involved in the Research? 

Only five out of 12 interventions included the target audience [34,35,36,38,39,40,42], while six included parents/caregivers [32,33,34,37,38,39,40,47,48]. Of these, one included the target audience only [35,36], one included parents/caregivers only [47,48], and one included both the target audience and parents/caregivers only [34]. In studies including the target audience in the participatory design of the intervention, students participated in assessing baseline data and in the strategic planning, implementation, and evaluation of the intervention. Additionally, students took part in focus groups or surveys to provide insight into baseline behaviors or perceptions of current institutional guidelines. When parents or caregivers were included in the participatory design, they provided feedback on proposed research plans, evaluated baseline data, and selected and implemented interventions. They also participated in focus groups to identify concerns, barriers, and current behaviors regarding childhood obesity. 

## 4. Discussion

This scoping review examined the characteristics of obesity prevention studies conducted with school-aged children in rural US communities. While many were school-based, only 12 of the 40 studies involved some form of community-engaged research. Community engagement is crucial for designing effective interventions [22], particularly for addressing significant public health issues, such as the obesity disparity identified among children in rural US communities [7,8]. Given that the rural population in the US is growing [5], there is an even greater need to develop interventions specifically designed to address the needs, preferences, and challenges faced by school-aged children in rural communities.

Despite the urban–rural childhood obesity disparities varying across countries [49], and the increasing trend of urbanization worldwide [50], community-engaged research remains crucial on a global scale. By actively engaging with communities, researchers can prioritize local knowledge and expectations, ensuring that interventions are culturally relevant and tailored to address the specific barriers of children in each context [51]. 

Rural communities often face unique challenges that should be considered when designing an obesity prevention intervention. These challenges include poverty [52,53,54,55], transportation issues [56], limited access to healthcare services and resources [53,57], readily available access to healthy, affordable foods [58,59], and safe places to play [60] in some, but not all communities. Community-engaged research can offer insight into the specific challenges faced by a community and offer unique suggestions for ways to overcome them.

Our review of community-engaged research in obesity prevention interventions in school-aged children living in rural communities identified only 12 studies that met the inclusionary criteria. While there is agreement that involving the community in research that affects them is important [23], it is not often reported in the literature. A systematic review examined both randomized clinical trials and non-randomized comparative effectiveness trials and found that out of a possible 371,159 trials, only 23 papers reported community-engaged research of some type, meaning that less than 1% of trials conducted in 2011–2016 included the community [61]. Therefore, our finding is not surprising. However, when examining publication dates of the articles included in the review reported here, there was a visible increase in the number of articles reporting utilizing community-engaged research (pre 2000 vs. 2000–current).

Recent research with teens living in rural communities revealed they perceived that being physically active was much easier in a rural community than locating healthy food options outside the home, an important insight when developing obesity prevention interventions for teens in rural US communities [62]. As an example, these findings suggest that obesity prevention interventions for these teens would need to emphasize maintenance of existing PA behaviors while helping them identify ways to find healthy food options away from home. The importance of community-engaged research in the creation of personally relevant interventions was also supported by insights obtained from 8- to 10-year-old Black/African American girls and parents who participated in an online obesity prevention intervention [63]; the findings clearly revealed that community-engaged research led to an intervention perceived as personally relevant and meaningful to both the girls and their parent/caregivers. These findings emphasize the need to conduct community-engaged research with the target audience in order to ensure that the interventions address issues perceived to be important to them. This is critical for the development of effective interventions in that personally relevant interventions are more likely to be appealing [64,65], leading to greater engagement and exposure to intervention content. Ultimately, greater engagement is more likely to result in behavior change, particularly when the intervention content addresses issues of personal importance.

Our review of the literature identified some improvements in health-related behaviors, environment, and psychosocial factors, such as improved knowledge or attitudes. However, only two out of seven studies [37,43,44] reported improvements in at least one anthropometric measure at the final follow up. Interestingly, community engagement was associated with improved dietary intake, with three out of five studies showing positive results [35,36,37,46]. These findings suggest that community engagement can be a helpful tool for promoting positive behavior change in youth. Future work is needed to investigate the types and levels of community engagement for promoting changes in specific youth behaviors like diet and physical activity. It could be that the type and level of community engagement may need to be tailored to the specific behavior being addressed. 

Our research aligns with findings from other studies that show inconsistent results regarding improvements in anthropometric measurements in response to obesity interventions. A recent systemic review found only 14 out of 33 interventions led to a reduction in BMI or BMI z-score [66]. However, this review also highlighted that involving key stakeholders improved intervention effectiveness. Similarly, a systematic review of obesity interventions in Hispanic children showed that those utilizing a community-based framework were more likely to reduce BMI compared to those not involving a community-based approach [67]. This review also reported that six out of ten studies improved fruit and vegetable consumption and reduced intake of calories, fat, and sodium. Notably, all six studies promoted the engagement of parents or the community in the activities. 

Based on these findings, we anticipated significant improvements in anthropometric measures and dietary habits due to the involvement of key stakeholders in the interventions analyzed. However, the lack of significant improvement in anthropometric measurements may suggest that achieving such changes in rural populations might be more challenging compared to other groups, such as Hispanic children [67]. 

Further research is needed to examine how to use community-engaged research to develop interventions designed to improve anthropometric measurements in children and youth living in rural communities. For example, work is needed to understand who to include when attempting to understand factors that impact body weight, body fat, and other anthropometric variables in youth, i.e., children or teens, parents/caregivers, and/or other members of the community. This research would contribute to greater insight regarding how to best utilize community engagement to understand these factors in youth and how to best utilize this information to develop interventions that “fit” within the rural context.

The underrepresentation of parents/caregivers in intervention design is another noteworthy finding. The need to involve parents or caregivers in interventions designed to impact the behavior of youth is well documented [68,69], particularly since parents are the gatekeepers of the home environment [70]. Parent’s choices and behaviors influence early childhood diet-related behaviors [71,72]. The involvement of parents or caregivers in changing a child’s behavior is further supported by the Family Systems Theory [73,74], which recognizes the family as a complex social system, with parents and children having an effect on each other’s behavior [75]. Thus, it is concerning that only three of the studies included in this review involved both parents or caregivers and the children targeted by the interventions. This suggested that future obesity prevention interventions for children in rural communities in the US should explore ways in which to involve parents or caregivers in intervention development or adaptation. By involving parents/caregivers in intervention development, we can create a more holistic approach that addresses the family unit as a whole.

Although there are many different ways and opportunities to conduct community-engaged research with children and their parents or caregivers, careful consideration should be given to the type of information needed, the status of intervention development, and or other considerations, such as the timeline, budget, and how much or little information already exists for a specific audience. Close consideration of the Key et al. categories [26] can facilitate the selection of the specific type of research needed to meet the project needs. For example, large national databases exist that can provide information in the beginning stages of research with a community to develop an understanding of their existing status regarding particular behaviors, such as the dietary status of particular groups [76] (community consultation). Other researchers may choose to conduct research with the community itself to identify particular issues from their perspective prior to intervention development (community consultation) [62]. In this type of research, interviews, focus groups, and other techniques, such as photovoice [77], can be useful techniques. To adapt an intervention or determine the relevance of an existing intervention for a particular population, the Delphi Technique could be used. A recent project convened a Community Advisory Board to review an existing intervention developed more than a decade ago; the Delphi Technique, combined with Key Point Summaries, resulted in high levels of both engagement and involvement [78] (community participation). There is no right or wrong way to conduct community-engaged research; however, it is important to perform some level of community-engaged research to understand issues from the communities’ perspective [26].

Finally, most of the studies used a quasi-experimental design, making it difficult to assess intervention effects. Future obesity prevention studies with school-aged children living in rural US communities should prioritize involving the target audience and their parents or caregivers in intervention design or adaptation, followed by assessment using a fully powered randomized design. In addition, future research should also explore the mechanisms of change within these interventions. This would help move the field forward by conducting robust research aimed at identifying strategies to reduce obesity risk in school-aged children in rural US communities. It would also help understand how interventions that influence behavior can inform future iterations and improve overall effectiveness.

This review highlights the need for future research on childhood obesity prevention in rural US communities to prioritize community-engaged approaches. Community-engaged research not only benefits intervention design but also fosters intervention sustainability [26,79,80,81]. By actively involving community members in research, we can help build trust and capacity within the rural communities and create a sense of ownership and investment in the intervention’s success. This can lead to increased community support and adoption of intervention, ultimately promoting long-term behavior change [79,80]. 

### Limitations

As in any study, there are limitations to this research. First, few of the studies included in this review provided a specific definition of rurality; instead, they simply reported that the school or community was located in a rural area. This lack of a standardized definition may have resulted in the inclusion of articles involving populations not traditionally classified as rural. Additionally, the term “rural” may have inadvertently excluded studies with a rural community that were not explicitly labeled as such. Second, although studies reported using theoretical frameworks, few specified how these theories were applied in intervention development. Thus, it was difficult to determine if the mentioned theories played a role in guiding the program development and implementation. Third, although we extracted data on the use and outcomes of community-engaged research in obesity prevention interventions tested with rural youth, we did not collect data on the outcomes of interventions that did not utilize community-engaged research. This information would be useful for those interested in determining the effectiveness of community-based interventions among rural youth. Fourth, comparing the outcomes of the interventions included in the review was challenging because of the variety of reported outcomes and the frequent reporting of outcomes not identified as primary or secondary. Further, the most common research design was quasi-experimental, leading to concerns regarding confounding variables [82]. Systematic reviews and meta-analyses of randomized controlled trials are needed to better understand the efficacy or effectiveness of community-engaged research in this context and to examine the type and intensity of community-engaged research with outcomes. 

## 5. Conclusions

This review revealed that community-engaged research is underutilized in obesity prevention interventions tested with school-aged children in rural U.S. communities. Of the 40 studies reviewed, only 12 involved some form of community engagement, and only three included both children and their parents or caregivers. Community-engaged research can be a valuable tool for understanding issues of personal importance to under-represented populations and developing interventions that reflect their needs, preferences, and expectations. With rural adolescents showing greater risks for obesity even while reporting high levels of PA, the underlying causes of obesity in this population are not well understood. Involving the target audience or relevant stakeholders, such as parents or caregivers, in the development and implementation of interventions for this under-served population may result in more tailored interventions addressing their barriers/concerns, ensuring cultural relevance, and leveraging community resources effectively. Further research examining the efficacy of obesity prevention interventions developed for children in rural US communities using community-engaged research is needed. Prioritizing community engagement may enable researchers to develop effective and sustainable solutions to address childhood obesity in rural US communities, ultimately empowering communities to take charge of their health and well-being.

## Figures and Tables

**Table 2:** Intervention characteristics.

Author(s) (Year of Publication)	Intervention Focus	Intervention Setting	Duration	Components	Theoretical Framework	Implementer
Askelson, N. M., et al. (2019) [42]	Diet	School—lunchroom	1 Academic Year	Cafeteria changes in food and prompts from foodservice staff	Behavioral Economics	Students, foodservice staff
Belansky, E. S., et al. (2006) [31]	Physical Activity and Diet	School	2 Years	Classroom based nutrition and PA lessons; 10 home visits that included fun activities and coaching techniques to motivate family members to make behavioral changes	Social Cognitive Theory, Piaget Cognitive Development Theory	Resource teachers, classroom teachers, and family advisor
Belansky, E. S., et al. (2013) [33]; Belansky, E. S., et al. (2009) [32]	Physical Activity and Diet	School	3 Academic Semesters	Implemented environmental and policy changes using AIM or SHI; AIM schools included 12 meetings led by trained, external facilitators with school task force while SHI schools had no external facilitation but were instead given a self-assessment and planning tool	Social Cognitive Theory	AIM—School task force led by external facilitator, SHI—School health team which included principal and a team of school staff
Canavera, M., et al. (2008) [34]	Physical Activity, Diet, and Watching less TV	School (PE class)	12 Weeks	Four modules that focused on PA, fruit/vegetable consumption, limiting television use, and replacing sweetened beverages with water	Social Cognitive Theory	Physical education or Health education teachers
de la Torre, et al. (2013) [43]; Sadeghi, et al. (2019) [44]	Physical Activity and Diet	School	3 Years	Nutrition education was provided on family nights and in school; the SPARK PA Program was implemented; families were provided with a monthly fruit and vegetable voucher worth USD 25/month; community art tools and strategies were used to engage community members	Social Cognitive Theory, Health Belief Model	Local health educators, local teachers, and the University of California Cooperative Extension
Donnelly, J. E., et al. (1996) [46]	Physical Activity and Diet	School	2 Academic Years	Used existing programming (Lunchpower program) to enhance PA, create grade-specific nutrition education, and a modified school lunch program	Not mentioned	Classroom teachers and cafeteria staff
Greening, L., et al. (2011) [37]	Physical Activity and Diet	School and Community	8 Months	Monthly nutrition and PA events for families; foodservice equipment changes; two 45 min PA sessions/week; incorporation of classroom nutrition lectures	Social Learning Theory	Dietitians, educators from the Department of Education, and school faculty
Gustafson, A. (2017) [35]; Gustafson, A. (2019) [36] for outcomes	Diet	Virtual	1 Academic Semester	Text messages were sent two times a week to encourage healthy food purchasing; weekly challenges were given as well	Social Cognitive Theory	Undergraduate students
Hawley, S. R., et al. (2006) [45]	Physical Activity and Diet	School	6 Weeks	Five 40 min sessions in PE class; community family event that provided a fitness option and education on nutrition and exercise	Principles of behavior change—Transtheoretical model	Unclear
Lynch et al. (2012) [48]; Eldridge (2016) [47]	Physical Activity and Diet	County Extension Office	8 Months	Ten, 90 min, face-to-face meetings covering healthy eating, PA, stress management, and effective parenting strategies; take-home materials such as handouts and recipes were given; control group received written information from USDA sources	Social Cognitive Theory, Health at Every Size approach, Social Marketing Theory	County agents
Schetzina, K.E., et al. (2009) [39]; Schetzina, K.E., et al. (2009) [40]	Physical Activity and Diet	School	18 Months	Nutrition services which included a series of interactive Go, Slow, and Whoa lesson plans; classroom health education lessons; increased PA during the school day; health screenings and referrals for students; counseling and psychological services; changes to the cafeteria menu and school environment; health promotion for staff; involvement of parents and community in promoting healthy behavior changes	Coordinated School Health Model	Classroom teachers, school health staff, school administration, parents
Smith, L. H., et al. (2014) [38]	Diet	School	30 Days	Promotional campaign which included a commercial flier, T-shirts, and posters; daily announcements about the benefits of limiting sweetened beverage consumption; distribution of nylon goody bags with promotional items; wellness presentations	Not mentioned	The Teen Advisory Council which consisted of teachers and students

AIM: Adapted Version of Intervention Mapping; PA: physical activity; PE: physical education; SHI: School Health Index.

**Table 3:** Researcher and community engagement in interventions.

Community Engagement Level	Example
Community-Informed	Statewide or national data used to determine intervention focus, content, mode
Community Consultation	Interviews or focus groups conducted to identify needs and to shape an intervention; researcher determines intervention focus (e.g., diet, physical activity)
Community Participation	Community advisory board to guide changes to a previously developed intervention
Community-Initiated	A community coalition approaches researchers to assist them with developing an intervention
Community-Based Participatory Research	Researchers and community jointly identify a need, determine how to address it, develop the intervention, assist in recruitment, evaluation and/or interpretation
Community-Driven/Community-Led	Community coalition identifies a problem, determines how to address it; may consult with a researcher for advice or questions, but community drives the research and makes decisions

Adapted from Key et al. [26].

**Table 4:** Stakeholder levels.

Stakeholder Level	Definition	Example
Level 1	Target audience	School-aged children
Level 2	Caregivers	Parents
Level 3	Intervention implementers	Teachers or school staff
Level 4	Community members not included in levels 1–3	City managers or local elected officials

**Table 5:** Participatory design of interventions.

Author(s) (Year of Publication)	Stakeholder Involvement	Community Engagement Level	Participants in Community Engaged Research	Stakeholder Level ^a^
Askelson, N.M., et al. (2019) [42]	Student group assisted in planning, implementing, and evaluating changes to lunchroom. Food service staff collaborated with students to review lunchroom assessments and jointly identify and plan changes.	CBPR	Students and food service staff	1, 3
Belansky, E.S., et al. (2006) [31]	Rural-based teachers and nutrition educators adapted lessons for culturally relevance	Community Participation	Teachers and nutrition educators	3
Belansky, E.S., et al. (2013) [33]; Belansky, E.S., et al. (2009) [32]	A steering committee consisting of school personnel made decisions on research design, school recruitment strategies, intervention plans, evaluation and dissemination plans, and other related matters. In AIM schools, a task force consisting of the principal, food service manager, parent(s), and school nurse evaluated the school environment and selected/implemented changes. In SHI schools, school staff were responsible for planning and implementing changes.	CBPR	Parents and school personnel including principal, foodservice manager, and school nurse	2, 3, 4
Canavera, M., et al. (2008) [34]	Focus groups consisting of parents and children were used to gather information on physical activity behaviors, watching television, and fruit/vegetable/water intake	Community Consultation	Parents and students	1, 2
de laTorre, A., et al. (2013) [43]; Sadeghi, B., et al. (2019) [44]	Research team conducted focused meetings with various community leaders; research team presented proposed research at town hall meetings to gather feedback; a Community Advisory Council comprising stakeholders from each community including representatives such as city managers, school superintendents, teachers, principals, school nurses, food service managers, local health facility representatives, religious leaders, community health outreach workers (promotores), and a representative from a major local supermarket was formed	Community Participation	Various community leaders including city managers, local elected officials, school superintendents and boards, teachers, religious leaders, local healthcare professionals, principals, school nurses, food service managers, local health facility representatives, community health outreach workers (promotores), and a representative from a major local supermarket	3, 4
Donnelly, J.E., et al. (1996) [46]	Kitchen staff helped plan meals to reflect Lunchpower; teachers helped develop and deliver nutrition education and physical activity program	Community Participation	Kitchen staff, teachers	3
Greening, L., et al. (2011) [37]	Focus groups were held with community residents to obtain their input on treatment activities that would complement the community’s activities. Parents completed a dietary habit questionnaire for their children and teachers incorporated health information in their lectures	Community-Informed	Parents, teachers, community members	2, 3, 4
Gustafson, A. (2017) [35]; Gustafson, A. (2019) for outcomes [36]	Student survey aimed to gather information about the adolescents’ food purchasing patterns, dietary intake, home food availability, and demographics	Community-Informed	Students	1
Hawley, S.R., et al. (2006) [45]	Community meetings were conducted to determine barriers to addressing youth obesity; director of the local recreation commission was interviewed; community church completed a survey	Community Consultation	Community members	4
Lynch, W.C., et al. (2012) [48]; Eldridge, G., (2016) [47]	Focus groups with parents were used to identify concerns regarding child obesity, interest in participating, and time constraints	Community Consultation	Parents	2
Schetzina, K.E., et al. (2009) [40]; Schetzina, K.E., et al. (2009) [39]	Focus groups with students, teachers, and parents were used to understand perceptions on institutional guidelines concerning nutrition and physical activity; a coalition consisting of educators, healthcare providers, parents, community members, and researchers designed the intervention and met monthly to discuss the results and make needed modifications	Community Participation	Educators, healthcare providers, parents, students, teachers and community members	1, 2, 3, 4
Smith, L.H., et al. (2014) [38]	A community survey was completed by community residents, school personnel, teens, and parents to identify health concerns; teachers and students were responsible for developing and delivering the intervention	CBPR	Students, parents, school staff, and community residents	1, 2, 3, 4

^a^ Level 1: school-aged children; Level 2: parents or caregivers; Level 3: those implementing the intervention, such as teachers or school staff; Level 4: the larger community not included in Levels 1–3; AIM: Adapted Version of Intervention Mapping; CBPR: community-based participatory research; SHI: School Health Index.

## Data Availability

Select articles and extraction categories are available from the corresponding author on an as-needed basis.

## Supplementary Materials

The following supporting information can be downloaded at: https://www.mdpi.com/article/10.3390/nu16142201/s1, Table S1: Medline Ovid search strategy.
