# Supplementary material for: Community-Based Participatory Obesity Prevention Interventions in Rural Communities: A Scoping Review
Source: Nutrients. 2024 Jul 10;16(14):2201. doi: 10.3390/nu16142201 (PMC11279648; doi:10.3390/nu16142201)
Supplement: Supplementary file 1 [file nutrients-16-02201-s001.zip › nutrients-3069382-supplementary.pdf]

Table S1. Medline Ovid search strategy.

No.	Search term(s)	Results
1	Obesity/ or Obesity, Morbid/ or Overweight/	230,895
2	(obes* or overweight* or "over-weight*").ti,ab,kw.	361,242
3	Weight Loss/	39,914
4	(weight* adj3 (loss* or lose* or losing* or reduc*)).ti,ab,kw.	136,848
5	exp Exercise/ or Dancing/	225,051
6	(exerci* or walk* or run* or jog* or swim* or danc*).ti,ab,kw.	672,664
7	(physical* adj3 activ*).ti,ab,kw.	138,689
8	"physical* activ*".ti,ab,kw.	136,179
9	exp Diet/	306,096
10	diet*.ti,ab,kw.	622,170
11	1 or 2	406,422
12	3 or 4 or 5 or 6 or 7 or 8 or 9 or 10	1,574,949
13	exp Child/ or Adolescent/	3,204,700
14	(pediatr* or paediatr* or child* or schoolchild* or boy* or girl* or kid or kids or youngster* or preteen* or tween* or teen* or adolescen* or juvenile* or youth*).ti,ab,kw.	2,107,592
15	(kindergar* or "kinder-gar*" or "elementary school*" or "primary school*" or "grammar school*" or "intermediate school*" or "middle school*" or "junior high*" or "high school*" or highschool* or "school-age*" or "K-12*" or "K12*" or "K through 12*" or "K-Twelve*" or "K through Twelve*").ti,ab,kw.	107,673
16	13 or 14 or 15	3,931,407
17	Rural Health/ or Hospitals, Rural/ or Rural Population/ or exp Rural Health Services/	101,583
18	(rural* or "non-urban*" or nonurban*).ti,ab,kw.	158,581
19	17 or 18	184,563
20	Primary Prevention/ or Health Promotion/ or Early Medical Intervention/ or Psychosocial Intervention/ or Internet-Based Intervention/ or exp Clinical Study/	1,138,234
21	prevention.fs,fx.	1,378,690
22	(prevent* or intervention* or "inter-vention*" or program*).ti,ab,kw.	3,247,036
23	(health* adj3 promot*).ti,ab,kw.	73,679
24	"health promotion*".ti,ab,kw.	37,025
25	(clinical* adj3 (trial* or study* or studies*)).ti,ab,kw.	704,425
26	20 or 21 or 22 or 23 or 24 or 25	5,299,868
27	Pediatric Obesity/	11,294
28	11 and 16	97,633
29	27 or 28	98,579
30	12 and 19 and 29	1,325
31	26 and 30	831
32	limit 31 to (English language and yr="2000 - Current")	761
